# Sleep and Wakefulness Correction in Different Seasons From Avicenna's Perspective

**DOI:** 10.5812/ircmj.7677

**Published:** 2013-03-05

**Authors:** Seyed Shahin Soltani, Bagher Minaii, Mehdi Besharat

**Affiliations:** 1 Faculty of Traditional Medicine, Shahid Beheshti University of Medical Sciences, Tehran, IR Iran; 2 Traditional Medicine and Material Medical Research Center, Shahid Beheshti University of Medical Sciences, Tehran, IR Iran; 3 Faculty of Medicine, Tehran University of Medical Sciences, Tehran, IR Iran; 4 Traditional Medicine Department, Faculty of Traditional Medicine of Tehran University of Medical sciences, Tehran, IR Iran; 5 Loqman-e-Hakim Hospital, Department of Infectious Diseases, Shahid Beheshti University of Medical Sciences, Tehran, IR Iran

**Keywords:** Sleep, Wakefulness, Avicenna, Ibn Sina

Dear Editor,

Heretofore, appreciable assays have been made by the authors of this journal to demonstrate the efforts of different Iranian traditional medicine scholars (1-4). Among them, Ibn Sina was a unique prodigy who has been known as Avicenna in west (980-1037AD). He was the author of more than 200 books on different branches of science such as medicine, one of his most important works being: Al-Qanun fi al-Tibb (The Canon of Medicine) (3, 4). One chapter of this textbook is dedicated to the study of sleep and wakefulness. The aim of the present letter is to briefly discuss sleep and wakefulness in relation to seasons, based on Avicenna's viewpoint. However, this evaluation can play an important role in health maintenance and prevention, because according to the Iranian traditional medicine, sleep in different seasons does not have the same recommendations for healthy individuals. Based on the principles of modern medicine, the amount of sleep is determined by several factors, among which the most important are the environment and the diurnal temperature (5). Traditional medicine beliefs support the theory of different amounts of sleep. Because of seasonal variance in temperature, the recommended amount of sleep should differ in various seasons, based on the rule of opposition in the traditional medicine, which claims that coldness should be treated with hotness, or vice versa and dryness with wetness, or vice versa (6). To support this, it is mandatory to explain some principles of Persian medicine. One of the important bases of traditional medicine in Iran is the prevention concept, which means keeping the body healthy by Sitteh-e-zarurieah (the six essentials),which include air, food and drink, sleep and wakefulness, movement and stillness, retention and vomiting, perturbations of mind, of which sleep and wakefulness play an essential role in correcting life style and preventing pathologic states. From the viewpoint of the Iranian traditional medicine, the role of medicine is to maintain the health at optimal levels, and to restore the health at time of illness (6, 7). Another fundament of Persian medicine is the Tabiat (nature of the body), which is an inherent ability of the human body that controls its homeostasis. The sleep mechanism, in the concept of Iranian traditional medicine, is the tendency of the Tabiat toward the inside of the body, which materializes into cooling of the surface of the body while warming the inside of the body. Therefore, it can be considered that sleep makes the body cold and wet. The arousal is the movement of the Tabiat towards the outside of the body that, by causing warmth, declines humidity and consequently dries and warms the body. This movement of the Tabiat makes the surface warm, but cools the inside of the body (6). According to Persian medicine, everything or object in the universe is composed of four elements with corresponding qualities (Air, with hot and wet, Water, with cold and wet, Fire, with hot and dry, and Earth, with cold and dry qualities). Another concept is the Mizaj (Temperament), which is a mean quality, representing the consequence of the interaction of these four elements. The temperament of spring is hot and wet, summer is hot and dry, autumn is cold and dry, and winter is cold and wet. On the other hand, based upon traditional medicine, the medical schemes are in the opposite direction. Therefore, all these schemes in spring should be applied to make coldness and dryness, coldness and wetness in the summer, hotness and wetness during autumn, and in winter, hotness and dryness(6, 7). Therefore, sleep, as a cold and wet making variable (6, 8) in the hot and dry summer, is more recommended than in the other seasons, to correct both the season temperament and strength recovery due to extreme exhaustion caused by summer heat. On the contrary, sleep is less recommended in winter compared to other seasons, because winter is a cold and wet season and the temperament of sleep is also cold and wet. Therefore, extreme sleeping causes excessive coldness in the surface of the body that will lead to specific disorders in this season and, in contrast, wakefulness in this season makes a corrective heat and dryness for counterbalancing the coldness and wetness of the environment. Again, sleep, with its wet temperament, due to the existence of humidity of the climate in spring, may be less recommended than in autumn. However, the case in autumn is different, because sleep, by making humidity, helps moderating the dryness of this season6.Consequently, we can suggest more sleep in autumn rather than during spring. In view of the above stated reasons, we propose the following order for the amount of recommended sleeping, with the purpose of keeping the body healthy, for healthy individuals in relation to seasons, starting from the longest to the shortest sleep duration:

Summer – autumn-spring-winter. This recommendation is the same for people within every age group(i.e. for children, in whom a greater amount of sleep is recommended than for the other age groups, sleep duration during summer should be longer than during the other seasons). Persian medicine assumes that if a factor in the human body has the ability to produce any of the four qualities (hotness, coldness, wetness and dryness),it will be able to change the state of the body toward health or illness. Therefore, changing the six essentials as in the relation between sleep and wakefulness, as a cold and wet maker phenomenon, is an important element in the prevention from several illnesses (9, 10) consequently, disobedience from the recommended amounts of sleep in different seasons may gradually lead to disease. For instance, lack of adequate sleep in summer may lead to more hours of arousal that may aggravate dryness and hotness effect of this season on the body or in autumn will lead to excessive dryness effect of the climate. On the other hand, sleep in excess in winter or spring may aggravate the unpleasant effect of humidity on the body in these two seasons. In conclusion, regarding the importance of having appropriate amounts of sleep along with maintaining body health, we can declare that from the point of view of the Iranian traditional medicine, sleep duration, in different situations, as in the case of seasonal variations of the year, is not recommended the same. However, stating a proper plan for different seasons may result in superior health maintenance and disease prevention. Future articles will investigate other interesting perspectives of traditional sleep medicine in relations with modern sleep medicine and will also emphasize how Avicenna's view may assist in revelation the idiopathic mechanisms of sleep related disorders.
